# Adjuvant bevacizumab for melanoma patients at high risk of recurrence: survival analysis of the AVAST-M trial

**DOI:** 10.1093/annonc/mdy229

**Published:** 2018-07-13

**Authors:** P G Corrie, A Marshall, P D Nathan, P Lorigan, M Gore, S Tahir, G Faust, C G Kelly, M Marples, S J Danson, E Marshall, S J Houston, R E Board, A M Waterston, J P Nobes, M Harries, S Kumar, A Goodman, A Dalgleish, A Martin-Clavijo, S Westwell, R Casasola, D Chao, A Maraveyas, P M Patel, C H Ottensmeier, D Farrugia, A Humphreys, B Eccles, G Young, E O Barker, C Harman, M Weiss, K A Myers, A Chhabra, S H Rodwell, J A Dunn, M R Middleton, AVAST-M Investigators, Paul Nathan, Paul Lorigan, Peter Dziewulski, Sonja Holikova, Udaiveer Panwar, Saad Tahir, Guy Faust, Anne Thomas, Pippa Corrie, Bhawna Sirohi, Charles Kelly, Mark Middleton, Maria Marples, Sarah Danson, James Lester, Ernest Marshall, Mazhar Ajaz, Stephen Houston, Ruth Board, David Eaton, Ashita Waterston, Jenny Nobes, Suat Loo, Gill Gray, Helen Stubbings, Martin Gore, Mark Harries, Satish Kumar, Andrew Goodman, Angus Dalgleish, Agustin Martin-Clavijo, Jerry Marsden, Sarah Westwell, Richard Casasola, David Chao, Anthony Maraveyas, Ernest Marshall, Poulam Patel, Christian Ottensmeier, David Farrugia, Alison Humphreys, Bryony Eccles, Renata Dega, Chris Herbert, Christopher Price, Murray Brunt, Martin Scott-Brown, Joanna Hamilton, Richard Larry Hayward, John Smyth, Pamela Woodings, Neena Nayak, Lorna Burrows, Virginia Wolstenholme, John Wagstaff, Marianne Nicolson, Andrew Wilson, Clare Barlow, Christopher Scrase, Timothy Podd, Michael Gonzalez, John Stewart, Martin Highley, Virginia Wolstenholme, Simon Grumett, Andrew Goodman, Toby Talbot, Kannon Nathan, Robert Coltart, Bruce Gee, Martin Gore, David Farrugia, Agustin Martin-Clavijo, Jerry Marsden, Christopher Price, David Farrugia, Kannon Nathan, Robert Coltart, Kannon Nathan, Robert Coltart

**Affiliations:** 1 Cambridge Cancer Centre, Cambridge University Hospitals NHS Foundation Trust, Cambridge, UK; 2 Warwick Clinical Trials Unit, University of Warwick, Coventry, UK; 3 Medical Oncology, Mount Vernon Hospital, Northwood, UK; 4 Department of Medical Oncology, Christie Hospital, Manchester, UK; 5 Royal Marsden Hospital NHS Trust, London, UK; 6 Oncology Research, Broomfield Hospital, Chelmsford, UK; 7 Oncology Department, Leicester Royal Infirmary, Leicester, UK; 8 Sir Bobby Robson Cancer Trials Research Centre, Freeman Hospital, Newcastle upon Tyne, UK; 9 Leeds Cancer Centre, St James’s University Hospital, Leeds, UK; 10 Weston Park Hospital, Academic Unit of Clinical Oncology, Sheffield, UK; 11 Cancer & Palliative Care, St. Helen’s Hospital, St. Helens, UK; 12 Oncology Department, Royal Surrey County Hospital, Guildford, UK; 13 Rosemere Cancer Centre, Royal Preston Hospital, Preston, UK; 14 Clinical Trials Unit, Beatson WOS Cancer Centre, Glasgow, UK; 15 Department of Clinical Oncology, Norfolk & Norwich University Hospital, Norwich, UK; 16 Guy’s & St. Thomas’ Hospital, Guy’s Cancer Centre, London, UK; 17 Velindre Cancer Centre, Cardiff, UK; 18 Exeter Oncology Centre, Royal Devon and Exeter Hospital, Exeter, UK; 19 St George’s Hospital, Cancer Centre, London, UK; 20 Cancer Centre, Queen Elizabeth Hospital, Birmingham, UK; 21 Sussex Cancer Centre, Royal Sussex County Hospital, Brighton, UK; 22 Cancer Centre, Ninewells Hospital, Dundee, UK; 23 Royal Free Hospital, London, UK; 24 Castle Hill Hospital, Cottingham, UK; 25 Academic Unit of Clinical Oncology, Nottingham University Hospitals NHS Trust, Nottingham, UK; 26 CRUK and NIHR Southampton Experimental Cancer Medicine Centre, Southampton University Hospitals NHS Foundation Trust, Southampton, UK; 27 Oncology Centre, Cheltenham General Hospital, Cheltenham, UK; 28 Oncology Department, James Cook University Hospital, Middlesbrough, UK; 29 Oncology Department, Poole Hospital, Dorset, UK; 30 Department of Oncology, University of Oxford, Oxford, UK; 31 Experimental Cancer Medicine Centre, Oxford, UK; 32 Melanoma Focus, Cambridge, UK; 33 Oxford NIHR Biomedical Research Centre, Oxford, UK

**Keywords:** melanoma, bevacizumab, adjuvant therapy

## Abstract

**Background:**

Bevacizumab is a recombinant humanised monoclonal antibody to vascular endothelial growth factor shown to improve survival in advanced solid cancers. We evaluated the role of adjuvant bevacizumab in melanoma patients at high risk of recurrence.

**Patients and methods:**

Patients with resected AJCC stage IIB, IIC and III cutaneous melanoma were randomised to receive either adjuvant bevacizumab (7.5 mg/kg i.v. 3 weekly for 1 year) or standard observation. The primary end point was detection of an 8% difference in 5-year overall survival (OS) rate; secondary end points included disease-free interval (DFI) and distant metastasis-free interval (DMFI). Tumour and blood were analysed for prognostic and predictive markers.

**Results:**

Patients (*n*=1343) recruited between 2007 and 2012 were predominantly stage III (73%), with median age 56 years (range 18–88 years). With 6.4-year median follow-up, 515 (38%) patients had died [254 (38%) bevacizumab; 261 (39%) observation]; 707 (53%) patients had disease recurrence [336 (50%) bevacizumab, 371 (55%) observation]. OS at 5 years was 64% for both groups [hazard ratio (HR) 0.98; 95% confidence interval (CI) 0.82–1.16, *P *=* *0.78). At 5 years, 51% were disease free on bevacizumab versus 45% on observation (HR 0.85; 95% CI 0.74–0.99, *P *=* *0.03), 58% were distant metastasis free on bevacizumab versus 54% on observation (HR 0.91; 95% CI 0.78–1.07, *P *=* *0.25). Forty four percent of 682 melanomas assessed had a *BRAF*^V600^ mutation. In the observation arm, *BRAF* mutant patients had a trend towards poorer OS compared with *BRAF* wild-type patients (*P *=* *0.06). *BRAF* mutation positivity trended towards better OS with bevacizumab (*P *=* *0.21).

**Conclusions:**

Adjuvant bevacizumab after resection of high-risk melanoma improves DFI, but not OS. *BRAF* mutation status may predict for poorer OS untreated and potential benefit from bevacizumab.

**Clinical Trial Information:**

ISRCTN 81261306; EudraCT Number: 2006-005505-64

Key Message:

The AVAST-M trial randomised 1343 patients with resected high risk cutaneous melanoma to receive either adjuvant bevacizumab (7.5 mg/kg i.v. 3 weekly for 1 year) or standard observation. Adjuvant bevacizumab significantly improved the disease-free interval but did not improve overall survival. BRAF mutation status predicted for poorer overall survival untreated and potential benefit from bevacizumab.

## Introduction

Angiogenesis is a host-dependent hallmark of cancer [1] and vascular endothelial growth factor (VEGF) is a key driver of angiogenesis [2]. VEGF is over-expressed in melanoma and high levels have been reported to be associated with poorer outcome [3–6]. Bevacizumab (Avastin^®^, F. Hoffman-La Roche AG, Basel, Switzerland) is a recombinant humanised monoclonal antibody to VEGF licensed for treatment of several common cancers, with modest activity reported in advanced melanoma [7]. Since VEGF is a relevant target in melanoma, we carried out a UK multi-centre, open-label, randomised controlled phase III trial of adjuvant bevacizumab versus standard surveillance in patients with resected cutaneous melanoma at high risk of recurrence.

The interim analysis of the AVAST-M trial when 1343 patients had been recruited and followed for more than 1 year showed a significant improvement in disease-free interval (DFI) with adjuvant bevacizumab [hazard ratio (HR) 0.83 (95% confidence interval [CI] 0.70–0.98), *P *=* *0.03] [8], which was well tolerated. We report the analysis of the primary overall survival (OS) end point, mandated when all surviving patients had been on study for at least 5 years.

## Methods

The study design, eligibility criteria, stratification variables and treatment schedules have been described previously in detail [8]. Briefly, patients at least 16-year old with histological confirmation of completely resected AJCC 7th edition stage IIB, IIC or IIIA–C cutaneous melanoma were eligible for the trial. Written informed consent was obtained for all patients. Multicentre Research Ethics Committee and regulatory approvals were obtained. Patients were followed up at least annually for 10 years after randomisation.

Eligible patients were randomly assigned to adjuvant bevacizumab (7.5 mg/kg i.v. infusion once every 3 weeks for 1 calendar year) or surveillance in a 1 : 1 ratio, stratified by primary tumour Breslow thickness, N stage, primary tumour ulceration status and patient sex. Randomisation occurred within 12 weeks of surgical resection and was carried out centrally using a computer minimisation algorithm held at the Warwick Clinical Trials Unit. This was an open-label trial.

### Biomarker analyses

At trial entry, plasma lactate dehydrogenase (LDH) was measured by local hospital laboratories for all patients. A patient was classed as having raised LDH if the value was above the upper level of normal (ULN) for their hospital. LDH was also measured centrally in plasma at baseline (pre-randomisation), 3 and 12 months from trial entry. VEGF and soluble VEGF receptor-1 (VEGFR1) were measured centrally by ELISA in both plasma and serum samples at baseline and then at 3, 12 and 24 months in exploratory patient cohorts. *BRAF* and *NRAS* mutation status were determined in archival tumour tissue using accredited methods.

### Statistical analysis

Patients (*n* = 1320; 660 patients per arm) were required to detect an 8% increase in the 5-year OS rate (primary end point) from 40% to 48% with 85% power and a 5% significance level, equating to an HR of 0.80. OS was defined as the time from date of randomisation until date of death from any cause, or censored at the last known date alive. Analysis was follow-up driven and pre-planned when all patients had been on study for 5 years.

Secondary end points were DFI, distant metastasis-free interval (DMFI), safety, toxicity and health-related quality of life (QoL). Adverse events were only collected during treatment and were reported previously [8]. Tertiary end points were to evaluate biological predictive and prognostic markers. DFI was defined as the time from date of randomisation until date of first tumour recurrence (including distant and locoregional recurrence), or date of death due to melanoma. DMFI was defined as the time from date of randomisation until date of first distant recurrent disease, or date of death due to melanoma. Survival from recurrence was defined as the time between the date of first tumour progression (in any site) and the date of death. Kaplan–Meier survival curves were constructed and a Cox proportional hazard model was used to obtain HRs and associated 95% CIs. Multivariable Cox regression models were used to adjust the treatment effect for stratification variables, to evaluate independent prognostic factors of OS and DFI and to assess treatment interactions. EORTC-QLQ-C30 QoL data were analysed by standardised area under the curve (AUC) and compared across trial arms using Wilcoxon rank sum tests. Mixed-effect models were used to assess whether VEGF and VEGFR1 levels changed over time or differed across trial arms. LDH levels measured over time were fitted as time-dependent continuous covariates in a Cox regression model.

Two-sided *P* values and 95% CIs are reported. All analyses were carried out on an intention-to-treat basis using the SAS statistical package.

## Results

Between 18 July 2007 and 29 March 2012, 1343 patients were randomised to either the bevacizumab (*N *=* *671) or observation (*N *=* *672) arms. Seven hundred fifty-three (56%) patients were male, their median age was 56 years (range 18–88 years), 364 (27%) patients had stage II melanoma, 195 (14%) had stage IIIA and 784 (59%) had stage IIIB/C disease. Sentinel lymph node biopsy (SLNB) was not mandated and 32% of patients in each arm underwent SLNB. Other baseline characteristics were similar between groups and were reported in full previously [8]. Six hundred eight two (51%) patients’ tumours were assessed for *BRAF* and *NRAS* mutation status; *BRAF* V600 and *NRAS* mutations were detected in 303 (44%) and 134 (20%) tumours tested.

With a median follow-up of 6.4 years, 515 (38%) patients had died: 254 (38%) of patients in the bevacizumab arm, 261 (39%) in the observation arm, 92% from metastatic melanoma on both arms. Seven hundred seven (53%) patients had melanoma recurrence: 336 (50%) in the bevacizumab arm, 371 (55%) in the observation arm. Of the 707 patients who had a recurrence, 117 (16%) patients had locoregional recurrence only, 359 (51%) had distant recurrence only and 231 (33%) had both locoregional and distant recurrence. One hundred twelve (16%) received an immune checkpoint inhibitor or targeted therapy as treatment for recurrence, totalling 55 (16%) on the bevacizumab arm and 57 (15%) on the observation arm (Table 1).

**Table 1.** Details of melanoma recurrence and associated treatment of recurrence

	Bevacizumab	Observation	Total
	(*N *=* *671)	(*N *=* *672)	(*N *=* *1343)
	*N* (%)	*N* (%)	*N* (%)
Patients with any recurrence	336 (50%)	371 (55%)	707 (53%)
Locoregional only	54 (16%)	63 (17%)	117 (16%)
Distant only	169 (50%)	190 (51%)	359 (51%)
Both locoregional and distant recurrence	113 (34%)	118 (32%)	231 (33%)
Treatment for any recurrence			
Immune checkpoint inhibitors/targeted therapy^a^	55 (16%)	57 (15%)	112 (16%)
Vemurafenib	27	34	61
Ipilimumab	19	17	36
Dabrafenib +/− trametinib	16	8	24
Ipilimumab + nivolumab	2	1	5
Pembrolizumab	2	2	4
Pazopanib	0	1	1
Vandetanib	1	0	1
Blinded ipilimumab, nivolumab or ipilimumab+nivolumab	0	2	2
Other systemic therapy	79 (24%)	97 (26%)	176 (25%)
Given as part of a clinical trial	9	19	28
Dacarbazine	56	59	115
Other cytotoxic chemotherapy	11	12	23
Other immunotherapy	3	5	8
Other biological agent	0	2	2
Surgery only	89 (26%)	119 (32%)	208 (29%)
Other (including radiotherapy)	66 (20%)	62 (17%)	128 (18%)
None	47 (14%)	36 (10%)	83 (12%)

a Patients could receive more than one line of treatment for recurrence; 98% patients receiving systemic therapy had distant metastatic disease.

**Figure 1.**
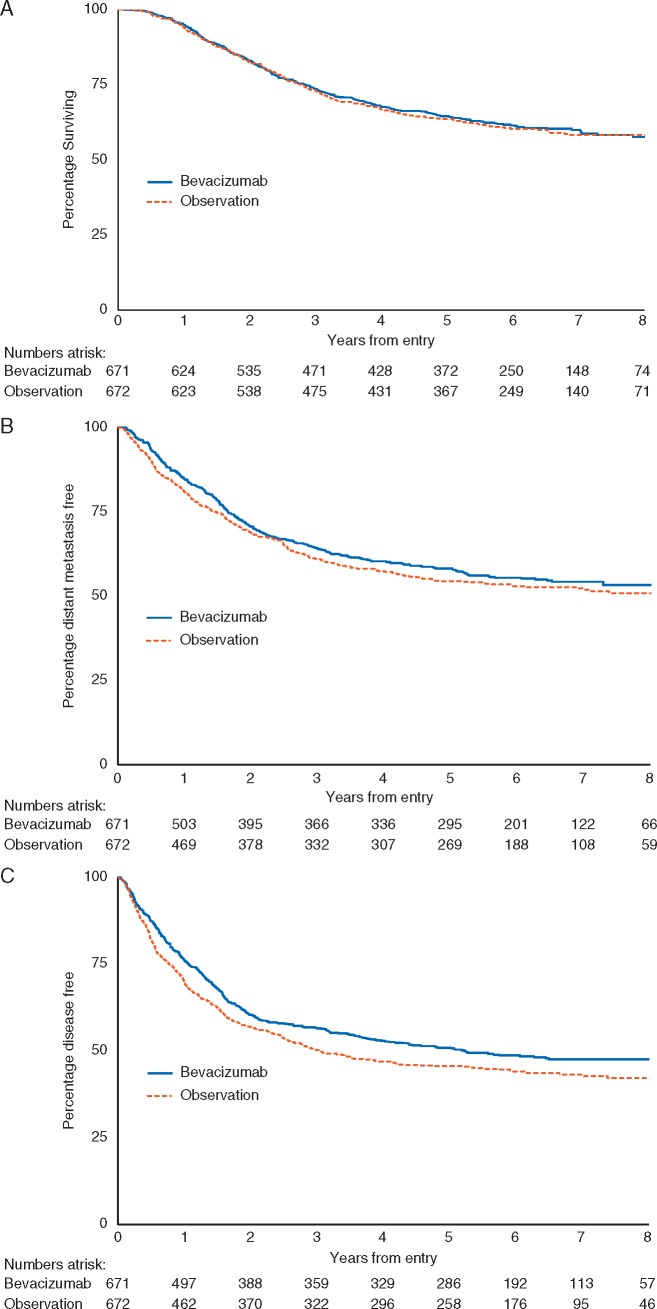
Overall survival (A), distant metastasis-free interval (B) and disease-free interval (C), by trial arm.

**Figure 2.**
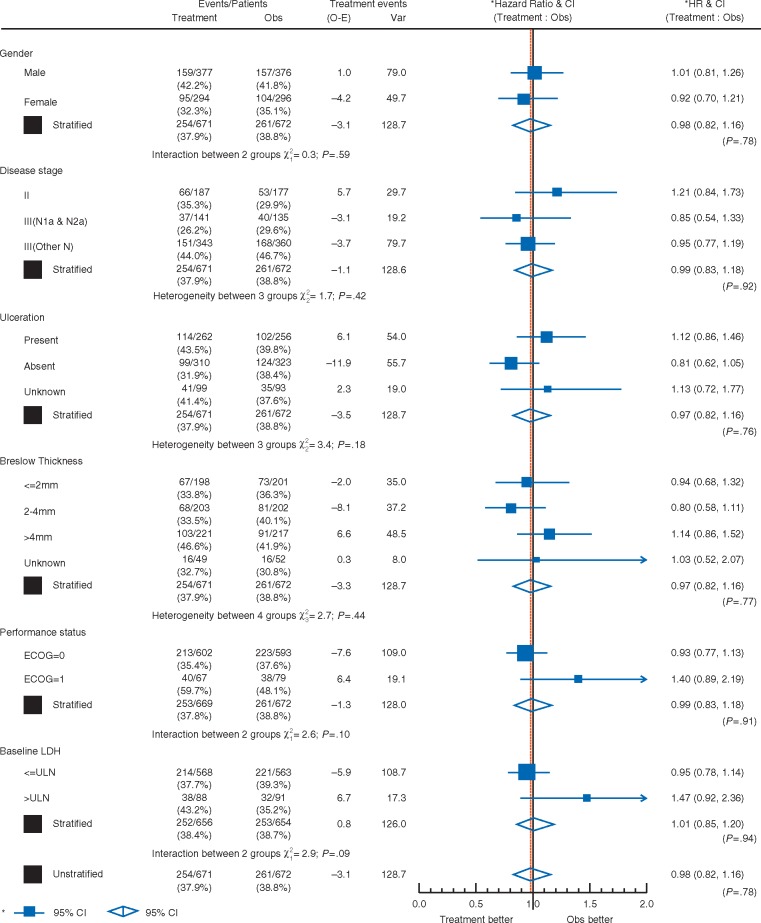
Hazard ratio plot of the treatment effect by prognostic factors for overall survival.

There was no significant difference in OS between trial arms (HR for bevacizumab = 0.98; CI 0.82–1.16; *P *=* *0.78, Figure 1A). The 5-year OS rate was 64% for both arms (CI 61%–68% for bevacizumab, 60%–67% for observation). Multivariate analysis identified disease stage, ECOG performance status, primary melanoma Breslow thickness and sex as independently prognostic of OS; trial arm remained non-significant (*P *=* *0.92; Table 2). There was no statistically significant interaction between any of these variables and trial arm (Figure 2).

**Table 2.** Multivariate analysis for overall survival for all trial patients and for the subgroup of patients for whom *BRAF* mutation status was assessed

	All trial patients			BRAF mutation status assessed			
	All trial patients	Deaths	Hazard ratio (95% CI)	BRAF mutant	BRAF WT	Deaths	Hazard ratio (95% CI)
	*N* (%)	*N* (%)		*N* (%)	*N* (%)	*N* (%)	
Total	1343			303	379		
Sex			*P* = 0.003				*P* = 0.19
Male	753 (56%)	316 (42%)	1.31 (1.10-1.57)	156 (51%)	230 (61%)	167 (43%)	1.18 (0.92-1.51)
Females	590 (44%)	199 (34%)	1.00	147 (49%)	149 (39%)	113 (38%)	1.00
Breslow thickness of primary melanoma			*P* = 0.0003				*P* = 0.004
<2.0 mm	399 (30%)	140 (35%)	1.00	126 (42%)	87 (23%)	83 (39%)	1.00
>2–4 mm	405 (30%)	149 (37%)	1.12 (0.89-1.42)	94 (31%)	108 (29%)	81 (40%)	1.16 (0.85-1.59)
>4 mm	438 (33%)	194 (44%)	1.53 (1.19-1.96)	65 (21%)	153 (40%)	101 (46%)	1.63 (1.16-2.27)
Unknown	101 (7%)	32 (32%)	0.75 (0.51-1.10)	18 (6%)	31 (8%)	15 (31%)	0.67 (0.38-1.17)
AJCC disease stage^a^			*P* < 0.0001				*P* < 0.0001
II	364 (27%)	119 (33%)	1.00	52 (17%)	117 (31%)	55 (33%)	1.00
IIIA	195 (15%)	41 (21%)	0.78 (0.53-1.48)	56 (19%)	29 (7%)	23 (27%)	1.00 (0.59-1.70)
IIIB	495 (37%)	210 (42%)	1.89 (1.47-2.44)	130 (43%)	147 (39%)	127 (46%)	2.18 (1.53-3.12)
IIIC	289 (21%)	145 (50%)	2.27 (1.74-2.96)	65 (21%)	86 (23%)	75 (50%)	2.40 (1.65-3.51)
ECOG performance status			*P* < 0.0001				*P* = 0.001
0	1195 (89%)	436 (36%)	1.00	269 (89%)	345 (91%)	240 (39%)	1.00
1	146 (11%)	78 (53%)	1.64 (1.29-2.10)	34 (11%)	33 (9%)	39 (58%)	1.75 (1.24-2.46)
Trial arm			*P* = 0.92				*P* = 0.83
Bevacizumab	671 (50%)	254 (38%)	1.01 (0.85-1.20)	132 (44%)	184 (49%)	128 (41%)	1.03 (0.81-1.30)
Observation	672 (50%)	261 (39%)	1.00	171 (56%)	195 (51%)	152 (42%)	1.00
BRAF status							*P* = 0.08
BRAF mutant				303 (100%)	0	129 (43%)	1.24 (0.97-1.59)
BRAF WT				0	379 (100%)	151 (40%)	1.00

a AJCC 7th edition.

The 5-year DMFI rate was 58% (CI 54%–62%) for the bevacizumab arm and 54% (CI 50%–58%) for the observation arm, but this was not statistically significantly different (HR = 0.91; CI 0.78–1.07; *P *=* *0.25, Figure 1B). The median DMFI for the bevacizumab arm was not reached (CI 7.3 years to limit not reached) and 9.6 years (CI 5.5–9.6 years) for the observation arm.

The significant improvement in DFI for those on the bevacizumab arm reported at the interim analysis was maintained over time (HR = 0.85; CI 0.74–0.99; *P *=* *0.03, Figure 1C) and persisted after adjustment for the stratification variables (HR = 0.86; CI 0.74–0.99; *P *=* *0.04). Patients receiving bevacizumab had a higher 5-year DFI rate (51%; CI 47%–55%) compared with the observation arm (45%; CI 42%–49%). The median DFI for patients in the bevacizumab arm was 63 months (CI, 44 months to limit not reached) and 37 months (CI 30–50 months) for those in the observation arm.

A high percentage (89%) of QoL forms were completed. There was no difference in overall QoL over the 5 years for the two trial arms: median standardised AUC for the QLQ-C30 global health scale was 81.7% [interquartile range (IQR) 69.8%–90.7%] for patients on the bevacizumab arm and 81.9% (IQR 68.6%–91.7%) on the observation arm (*P *=* *0.52).

**Figure 3.**
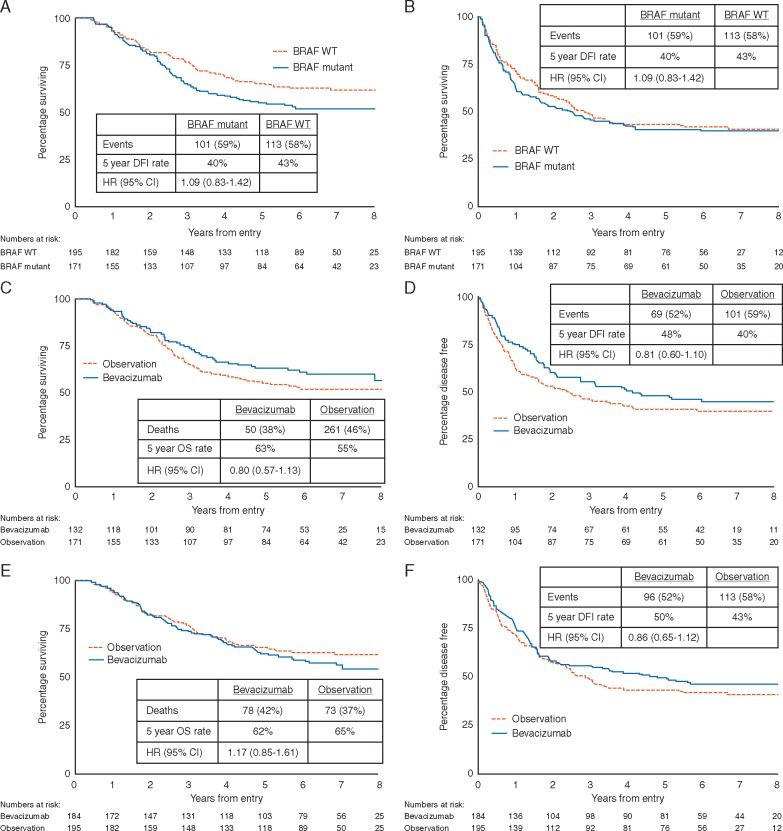
Overall survival (A) and disease-free interval (B) by *BRAF* status for the observation arm patients only; Overall survival and disease-free interval by trial arm for *BRAF* mutant patients (C and D); Overall survival and disease-free interval by trial arm for *BRAF* wild type patients (E and F).

In the observation arm, *BRAF* mutant melanoma patients had poorer OS compared with *BRAF* wild-type melanomas (*P *=* *0.06, Figure 3A). Overall, this effect was similar after adjustment for disease stage, ECOG performance status, primary melanoma Breslow thickness and sex (*P *=* *0.08, Table 2). A trend for improved OS with bevacizumab was only evident for the patients with *BRAF* mutant melanomas (HR = 0.80; CI 0.57–1.13; *P *=* *0.21, Figure 3C) and not seen in the patients with *BRAF* wild-type melanomas (HR = 1.17; CI 0.82–1.61; *P *=* *0.34, Figure 3E). *BRAF* mutant patients received more checkpoint inhibitors/targeted therapy at recurrence (22% versus 9%, supplementary Table S1, available at *Annals of Oncology* online), but the benefit from bevacizumab was evident for DFI as well as OS (Figure 3D).

At trial entry 179 (13%) patients had plasma LDH levels above the hospital reported ULN. Baseline LDH was not found to be prognostic of DFI (HR = 1.01, CI 0.81–1.25, *P *=* *0.97), DMFI (HR = 1.10, CI 0.88–1.39, *P *=* *0.40) or OS (HR = 1.05; CI 0.81–1.35; *P *=* *0.73). LDH measurements across three time-points—baseline, 3 months and 12 months—were also assessed. After fitting LDH as a time-dependent continuous covariate, LDH was still not found to be prognostic of DFI (HR = 1.00, CI 0.96–1.03 per 50 unit increase, *P *=* *0.81), DMFI (HR = 1.01, CI 0.97–1.05 per 50 unit increase, *P *=* *0.56) or OS (HR = 1.02, CI 0.98–1.06 per 50 unit increase, *P *=* *0.36).

Patients (*N *=* *414; 198 in the bevacizumab arm, 216 in the observation arm) had VEGF and VEGFR1 plasma and serum measurements at baseline and serially over the 2 years from randomisation (supplementary Figure S1, available at *Annals of Oncology* online). Neither baseline plasma nor serum VEGF were prognostic factors for OS (HR = 1.02, CI 0.95–1.10 per 50 unit increase, *P *=* *0.53 for plasma; HR = 1.03, CI 0.98–1.07 per 50 unit increase, *P *=* *0.21 for serum). Serum, but not plasma, VEGF levels significantly fell over time in the bevacizumab-treated patients compared with observation (*P *<* *0.0001 and *P *=* *0.58, respectively). Neither baseline plasma nor serum VEGFR1 were prognostic factors for OS (HR = 0.85, CI 0.58–1.25 per 50 unit increase, *P *=* *0.41 for plasma; HR = 0.86, CI 0.60–1.22 per 50 unit increase, *P *=* *0.40 for serum). Plasma VEGFR1 levels increased during bevacizumab treatment compared with observation (*P *<* *0.001). However, VEGFR1 serum results did not vary over time (*P *=* *0.75) or by trial arm (*P *=* *0.92).

## Discussion

AVAST-M represents the largest trial in a melanoma patient population evaluating angiogenesis inhibition. This survival analysis was pre-planned when all patients had been on study for 5 years. With longer follow-up, the trial has confirmed the interim finding that adjuvant bevacizumab improved DFI [8]. The HR of 0.85 favouring bevacizumab is comparable to the event-free survival HR of 0.86 reported for adjuvant interferon in a recent meta-analysis [9]. However, while for adjuvant interferon this HR translated into a small OS benefit, this was not the case for bevacizumab. The conditional power for futility of the primary outcome of OS was less than 10%. Therefore adjuvant bevacizumab cannot be recommended as a standard adjuvant therapy after resection of melanoma at high risk of recurrence.

The 64% 5-year OS rate for both observation and treatment arms of the AVAST-M trial was notably higher than predicted when the trial was designed. The original statistical premise was based on the results of the UK AIM High trial, which recruited patients with similar demographics between 1995 and 2000 [10]. Since then, improvements in healthcare and more accurate staging have contributed to an upward trend in melanoma patient survival [11]. The step change is evident in observation arms of other adjuvant melanoma trials: EORTC 18991 recruited stage III patients only between 2000 and 2003 and had a 7-year OS of 46% [12], while the 5-year OS rate in the EORTC 18071 trial which recruited similar patients between 2008 and 2011 was 54% [13]. The AVAST-M observation arm carried out even better, although one quarter of patients had lower risk stage II disease.

During the time that AVAST-M was recruiting, MAP kinase inhibitors and immune checkpoint inhibitors were approved for treatment of metastatic melanoma and are now standard of care. Only 16% of patients taking part in AVAST-M received these drugs at recurrence and the proportions were equal between the two trial arms, so we can be confident that treatment at recurrence cannot explain the lack of survival benefit from bevacizumab reported here. Central gene mutation testing for just over half of recruited patients identified *BRAF* and *NRAS* mutation rates of 44% and 20%, respectively. These proportions reflect those in metastatic melanoma populations, suggesting stability over time.

We used this large-scale adjuvant trial to explore potential prognostic and predictive biomarkers. Although LDH may be of prognostic value in metastatic disease, this was not the case after melanoma resection. Our study represents the most comprehensive analysis of angiogenesis biomarkers associated with a melanoma patient cohort conducted to date, but has not identified any immediate clinical value in measuring VEGF or VEGFR1 after melanoma surgery. Other circulating factors associated with angiogenesis [14] could be considered in future melanoma trials evaluating angiogenesis inhibitors.

The most common melanoma genetic mutation, *BRAF*, is a near-perfect biomarker predictive of sensitivity to BRAF targeted therapies in both advanced and high-risk resected melanoma [15]. Its role as a prognostic marker in each of these disease stages is, however, controversial [16]. Survival differences according to *BRAF* mutation status reported for patient cohorts after resection of primary melanomas have been inconsistent [17–19]. In our study, we saw a trend in poorer OS for *BRAF* mutant patients compared with *BRAF* wild-type patients, although this did not reach statistical significance. We also identified a trend towards enhanced OS from adjuvant bevacizumab limited only to the subgroup of patients with *BRAF* mutated tumours. *BRAF* mutation status was recently reported to describe populations with differing OS after immune checkpoint inhibitors [20]. *BRAF* V600E is pro-angiogenic in several human tumour models [21, 22], while VEGF has wider regulatory function beyond angiogenesis, including on immune cells [23–25]^.^ Exploratory studies combining bevacizumab with ipilimumab [26] or atezolizumab [27, 28] have reported early efficacy signals. Our findings raise the hypothesis that combining bevacizumab with adjuvant immune checkpoint inhibitors may benefit high-risk *BRAF* mutant melanoma patients, who in our study had a poorer prognosis than patients with tumours lacking the mutation. 

## Supplementary Material

Supplementary Figure S1

Supplementary Table S1
