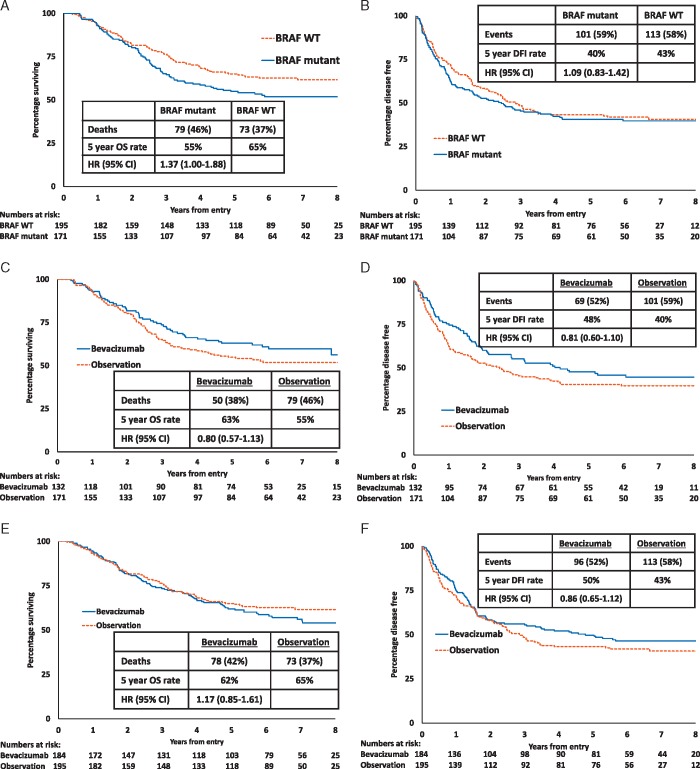# Adjuvant bevacizumab for melanoma patients at high risk of recurrence: survival analysis of the AVAST-M trial

**DOI:** 10.1093/annonc/mdz237

**Published:** 2019-08-20

**Authors:** P G Corrie, A Marshall, P D Nathan, P Lorigan, M Gore, S Tahir, G Faust, C G Kelly, M Marples, S J Danson, E Marshall, S J Houston, R E Board, A M Waterston, J P Nobes, M Harries, S Kumar, A Goodman, A Dalgleish, A Martin-Clavijo, S Westwell, R Casasola, D Chao, A Maraveyas, P M Patel, C H Ottensmeier, D Farrugia, A Humphreys, B Eccles, G Young, E O Barker, C Harman, M Weiss, K A Myers, A Chhabra, S H Rodwell, J A Dunn, M R Middleton

Ann Oncol 2018; 29(8): 1843–1852 (doi: 10.1093/annonc/mdy229)

In the original article, there was an error in Figure 3. The figure has now been corrected as below in the online version of the article.

**Figure 3.**