# Supplementary figures and images for: Adjuvant bevacizumab for melanoma patients at high risk of recurrence: survival analysis of the AVAST-M trial
Source: Ann Oncol. 2018 Jul 13;29(8):1843–52. doi: 10.1093/annonc/mdy229 (PMC6096737; doi:10.1093/annonc/mdy229)

***Supplementary Figure 1 (online only):***

1. B)


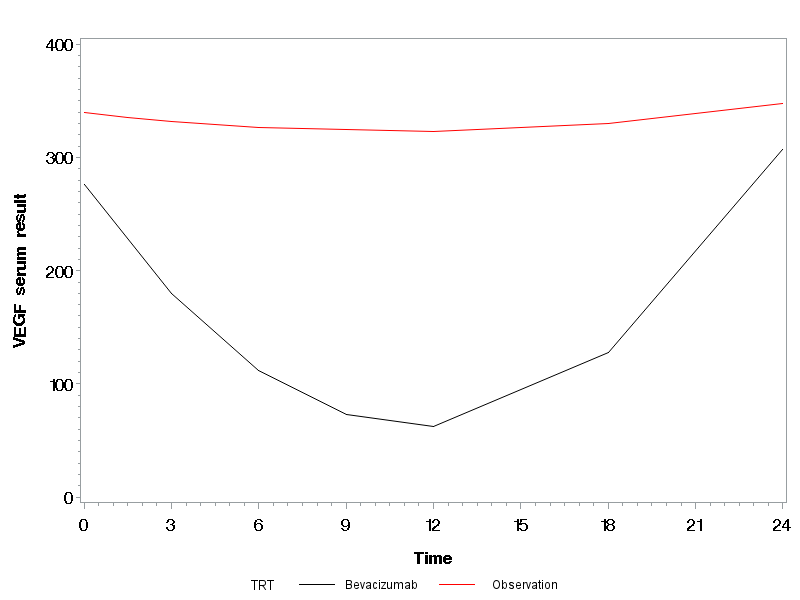

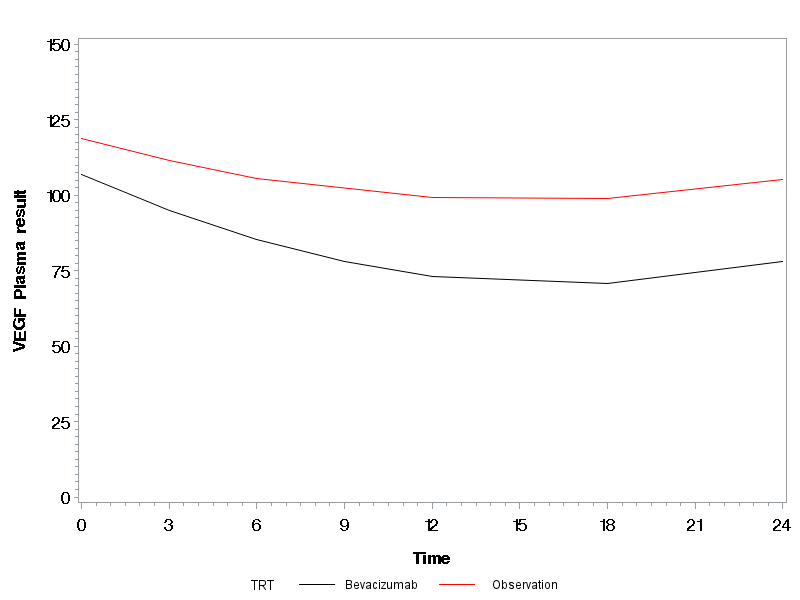


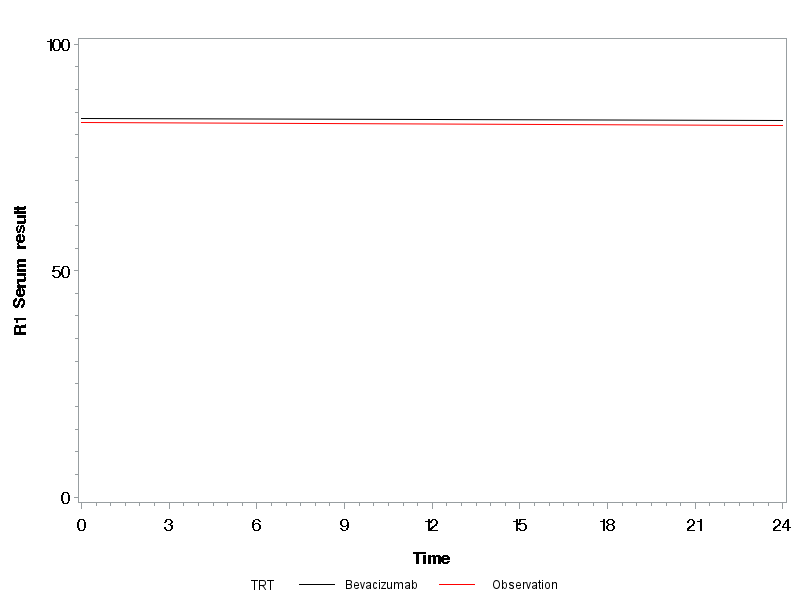
 C) D)
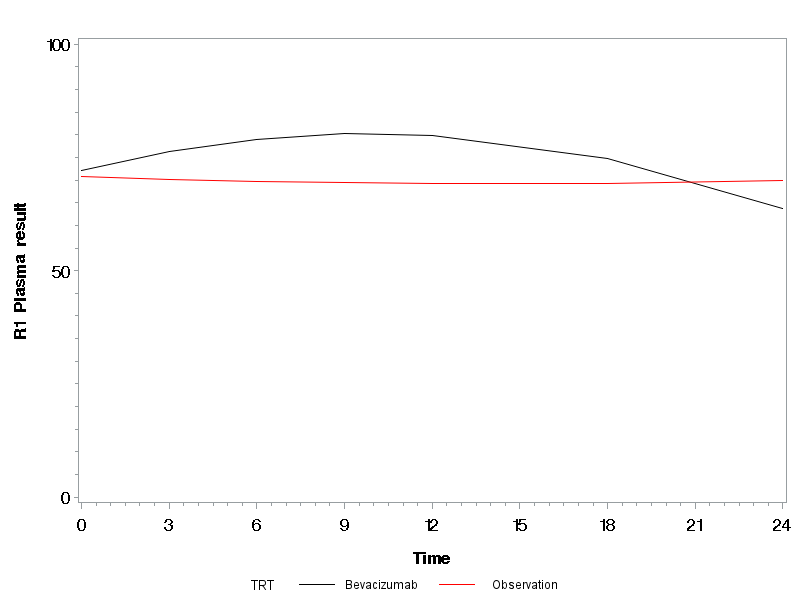

Supplement: Supplementary Figure S1 [file mdy229_supplementary_figure_1.docx]
