# Supplementary material for: Adjuvant bevacizumab for melanoma patients at high risk of recurrence: survival analysis of the AVAST-M trial
Source: Ann Oncol. 2018 Jul 13;29(8):1843–52. doi: 10.1093/annonc/mdy229 (PMC6096737; doi:10.1093/annonc/mdy229)
Supplement: Supplementary Table S1 [file mdy229_supplementary_table_1.docx]

**Supplementary Table 1 (online only): Details of recurrence and associated treatment for recurrence by *BRAF* mutation status**

|  |  | ***BRAF* mutant** | ***BRAF* WT** |
| --- | --- | --- | --- |
|  |  | *N* (%) | *N* (%) |
| **Total** | | 303 | 379 |
| **No recurrence** | | 133 (44%) | 170 (45%) |
| **Patients with any recurrence** | | 170 (56%) | 209 (55%) |
| **Patient with a distant recurrence** | | 150 (50%) | 168 (44%) |
| **Treatment for any recurrence** | |  |  |
|  | **Immune checkpoint inhibitors/targeted therapy** | 37 (22%) | 18 (9%) |
|  | **Other systemic therapy** | 45 (27%) | 56 (27%) |
|  | **Given as part of a clinical trial** | 4 | 8 |
|  | **Dacarbazine** | 32 | 41 |
|  | **Other Cytotoxic chemotherapy** | 6 | 6 |
|  | **Other immunotherapy** | 2 | 1 |
|  | **Other Biological agent** | 1 | 0 |
|  | **Surgery only** | 50 (29%) | 67 (32%) |
|  | **Other (including radiotherapy)** | 24 (14%) | 43 (20%) |
|  | **None** | 14 (8%) | 25 (12%) |
